# The effect of unilateral disruption of the centrifugal visual system on normal eye development in chicks raised under constant light conditions

**DOI:** 10.1007/s00429-016-1279-9

**Published:** 2016-08-17

**Authors:** Christopher Mark Dillingham, Jeremy Andrew Guggenheim, Jonathan Thor Erichsen

**Affiliations:** 1 School of Optometry and Vision Sciences, Cardiff University, Maindy Road, Cardiff, CF24 4HQ Wales, UK; 2 Institute of Neuroscience, Trinity College Dublin, Lloyd Building, College Green, Dublin 2, Ireland

**Keywords:** Refractive error, Ectopic area, Hyperopia, Anisometropia, Circadian, Myopia, Hindbrain

## Abstract

The centrifugal visual system (CVS) comprises a visually driven isthmic feedback projection to the retina. While its function has remained elusive, we have previously shown that, under otherwise normal conditions, unilateral disconnection of centrifugal neurons in the chick affected eye development, inducing a reduced rate of axial elongation that resulted in a unilateral hyperopia in the eye contralateral to the lesion. Here, we further investigate the role of centrifugal neurons in ocular development in chicks reared in an abnormal visual environment, namely constant light. The baseline ocular phenotype of constant light-reared chicks (*n* = 8) with intact centrifugal neurons was assessed over a 3-week post-hatch time period and, subsequently, compared to chicks raised in normal diurnal lighting (*n* = 8). Lesions of the isthmo-optic tract or sham surgeries were performed in another seventeen chicks, all raised under constant light. Ocular phenotyping was performed over a 21-day postoperative period to assess changes in refractive state (streak retinoscopy) and ocular component dimensions (A-scan ultrasonography). A pathway-tracing paradigm was employed to quantify lesion success. Chicks raised in constant light conditions with an intact CVS developed shallower anterior chambers combined with elongated vitreous chambers relative to chicks raised in normal diurnal lighting. Seven days following surgery to disrupt centrifugal neurons, a significant positive correlation between refractive error asymmetry between the eyes and lesion success was evident, characterized by hyperopia in the eye contralateral to the lesion. By 21 days post-surgery, these contralateral eyes had become emmetropic, while ipsilateral eyes had developed relative axial hyperopia. Our results provide further support for the hypothesis that the centrifugal visual system can modulate eye development.

## Introduction

Reciprocal anatomical connections are a common feature of neural pathways mediating sensory processing and, in particular, visual processing. In sauropsids, i.e., non-mammalian amniotes, the dominant (retino-tectal) visual pathway has extensive feedback projections from the optic tectum (the sauropsid homologue of the superior colliculus) to the visual isthmic complex, while, in the dominant mammalian (retino-thalamic) visual pathway, the primary visual cortex has major feedback projections to the lateral geniculate nucleus (LGN). In both cases, these projections are topographically organized. In mammals, this principle breaks down, however, when one considers feedback to retinal ganglion cells. Although occupying a peripheral position outside of the brain, the eye actually begins its development within the diencephalon and only, subsequently, emerges from the brain, remaining connected via the optic nerve. Yet, the LGN does not provide any input to the retinal ganglion cells that give rise to the axons within the optic nerve. In marked contrast, a rhombencephalic centrifugal visual system (CVS) in sauropsids does receive a direct topographic input from the optic tectum pathway, forming a closed loop neural circuit that is defined by an efferent projection from the hindbrain to the retina.

The CVS is particularly enlarged and well defined in birds (Gutierrez-Ibanez et al. 2012; for a recent review, see Wilson and Lindstrom 2011), and for this reason, birds have been the focus of the majority of functional, anatomical and physiological studies of the CVS, e.g., in the chicken (Miles 1972; Clarke et al. 1976; Marin et al. 1990), pigeon (Cowan and Powell 1962; McGill et al. 1966; Galifret et al. 1971; Woodson et al. 1991) and Japanese quail (Uchiyama and Ito 1993; Uchiyama et al. 2012). In the chicken, layers 9/10 of the optic tectum transmit predominantly (but not exclusively) visual information to the ipsilateral isthmo-optic nucleus (ION; Holden 1968) and, potentially, neurons of the surrounding ectopic area (EA; Hayes and Webster 1981; Woodson et al. 1995). In many bird species, the centrifugal pathway comprises two parallel, but distinct projections within the isthmo-optic tract (IOTr; Cowan et al. 1961). The first arises from the ION, whose restricted efferent fibers terminate in the retina upon single isthmo-optic target cell (IOTCs) in a distribution confined to the ventral half of the contralateral retina. In turn, these IOTCs send excitatory intra-retinal axons to retinal ganglion cells (RGCs) throughout the retina (Catsicas et al. 1987), where they modulate RGC gain. The second arises from centrifugal ectopic area (EA) neurons, whose divergent efferent fibers branch extensively in the retina to terminate upon many displaced retinal ganglion cells (dRGCs) over a wide area of the ventral retina (Maturana and Frenk 1965; Woodson et al. 1995; Lindstrom et al. 2009). In turn, dRGCs provide the sole visual input to the nucleus of the basal optic root of the accessory optic system (Fite et al. 1981) and nucleus lentiformis mesencephalic of the pretectum (Wylie et al. 2015), thus forming the sensory drive for the visuomotor responses that underlie stabilization of gaze. In addition, a small population of centrifugal ectopic neurons terminate in the ipsilateral retina (Clarke and Cowan 1975); however, as yet neither the neural pathway by which the axons of these neurons reach the ipsilateral retina nor their intra-retinal target(s) are known. Thus, for the purpose of this study, an assumption was made that the fibers of this projection reach their ipsilateral targets simply by remaining uncrossed in the optic chiasm. That said, alternative explanations exist, including the possibility that ipsilateral ectopic axons decussate within the midbrain with axons of the trochlear nucleus before re-crossing at the optic chiasm to reach their intended destination.

The chick has been widely used as a model for investigations into the mechanisms of emmetropization, the term given to the process by which neo-natal refractive error is reduced from hyperopia towards zero (Wallman and Adams 1987; Schaeffel and Howland 1991). Emmetropization is generally accepted to be an active process, guided by visual experience, which is in turn aided by genetically determined, non-visually guided growth (Troilo and Wallman 1991; Wildsoet and Wallman 1995; Chen et al. 2011). The effects of constant light on emmetropization are particularly severe in chicks (Weiss and Schaeffel 1993; Li et al. 1995; Wahl et al. 2015). Flattening of the cornea, accompanied by lens thinning, results in a relative shortening of the anterior chamber. The development of such anterior segment effects, in spite of an associated vitreous chamber elongation, results in severe hyperopia (e.g., constant light: +18.20 dioptres (D); diurnal light: +2.80 D; after 11 weeks of treatment; Li et al. 1995). However, if chicks are returned to normal diurnal lighting conditions within 11 weeks of constant light rearing, refractive recovery and normal emmetropization occur (Wahl et al. 2015).

When the retina is disconnected from the brain through optic nerve section (Troilo et al. 1987), eyes retain the ability to respond to visual cues (Wildsoet and Wallman 1995; Wildsoet 2003). Indeed, eyes that are disconnected in this way are still able to respond locally to defocus or form deprivation (FD) that is confined to local retinal regions (Wallman et al. 1987; Miles and Wallman 1990). It is important to note that severing the optic nerve disrupts not only the efferents of RGCs to the primary visual centers of the brain but also the axons of the CVS that terminate within the retina. We have previously reported that disruption of centrifugal neurons, while preserving retinal ganglion cell axons, has comparable effects to those reported following optic nerve section: both manipulations result in vitreous chamber-dependent hyperopia (Troilo et al. 1987; Wildsoet and Wallman 1995; Wildsoet 2003; Dillingham et al. 2013). Specifically, following disruption of centrifugal axons but leaving RGC axons within the optic nerve intact, chicks developed an initial hyperopia in the eye contralateral to the lesion. Crucially, however, unlike the persistent disruption of emmetropization observed following optic nerve section, emmetropia was re-established by the end of the 3-week observation period (Dillingham et al. 2013).

**Fig. 1:**
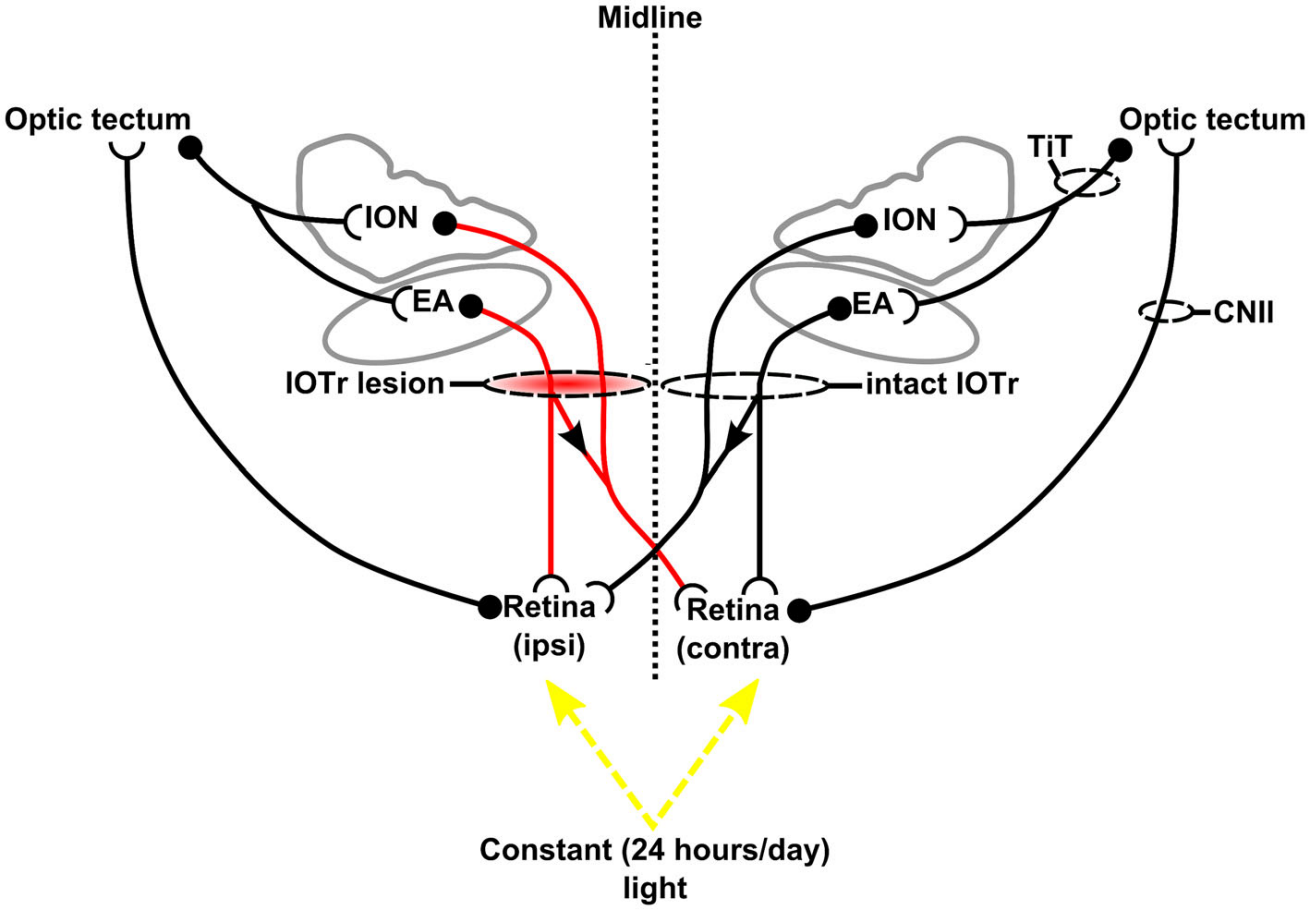
A schematic illustration of the experimental paradigm. Refractive and ocular component development were assessed over a 21-day post-surgical period during which chicks were reared in constant light conditions. A unilateral, left hemisphere lesion of the isthmo-optic tract (IOTr) was combined with constant light rearing. The isthmo-optic nuclei (ION) and the surrounding ectopic area (EA) receive input from the retina, indirectly via the optic tectum. Unilateral lesion of the IOTr disrupts contralateral ION and EA centrifugal pathways as well as the EA projection to the ipsilateral retina (*red* pathways), while ipsilateral EA inputs to this retina remain intact. In contrast, contralateral ION and EA inputs to the retina ipsilateral to the lesion are not affected by the lesion, but ipsilateral EA inputs to the ipsilateral retina are disrupted. Abbreviations: CNII, optic nerve; TiT, tecto-isthmic tract

Thus, while emmetropization is generally accepted to be predominantly driven by intra-ocular mechanisms (Wildsoet and Schmid 2000), CVS lesion and optic nerve section studies in the chick suggest that higher brain centers may play an important modulatory role (Troilo and Wallman 1991; Wildsoet and Wallman 1995). Indeed, such evidence strongly indicates that a neural connection between the eye and the brain is a prerequisite for (at least) fine-tuning refractive development. To further characterize centrifugal modulation of ocular growth, we report here upon the effects of disrupting centrifugal efferents to the eye in chicks raised under constant light conditions (Fig. 1).

## Methods

### Animals and treatments

A total of 25 Shaver Black chicks (a cross between Rhode Island Red and Barrack Rock strains), obtained as fertilized eggs from a hatchery specialized for biomedical research (Henry Stewart Ltd.), were used in this study. Once hatched, the chicks were transferred to a custom made, temperature controlled (25–27 °C) brooding pen for the first week, before being moved to a larger pen heated by an overhead lamp for the remainder of the study. Chicks were raised from hatch in constant light conditions (24 h/day). The illumination in the brooder and floor pen was 250–300 lx. Food and water were available ad libitum, with the exception of 1–2 h prior to induction of general anesthesia. During this brief period, chicks were not provided with food but had unlimited access to water.

**Fig. 2:**
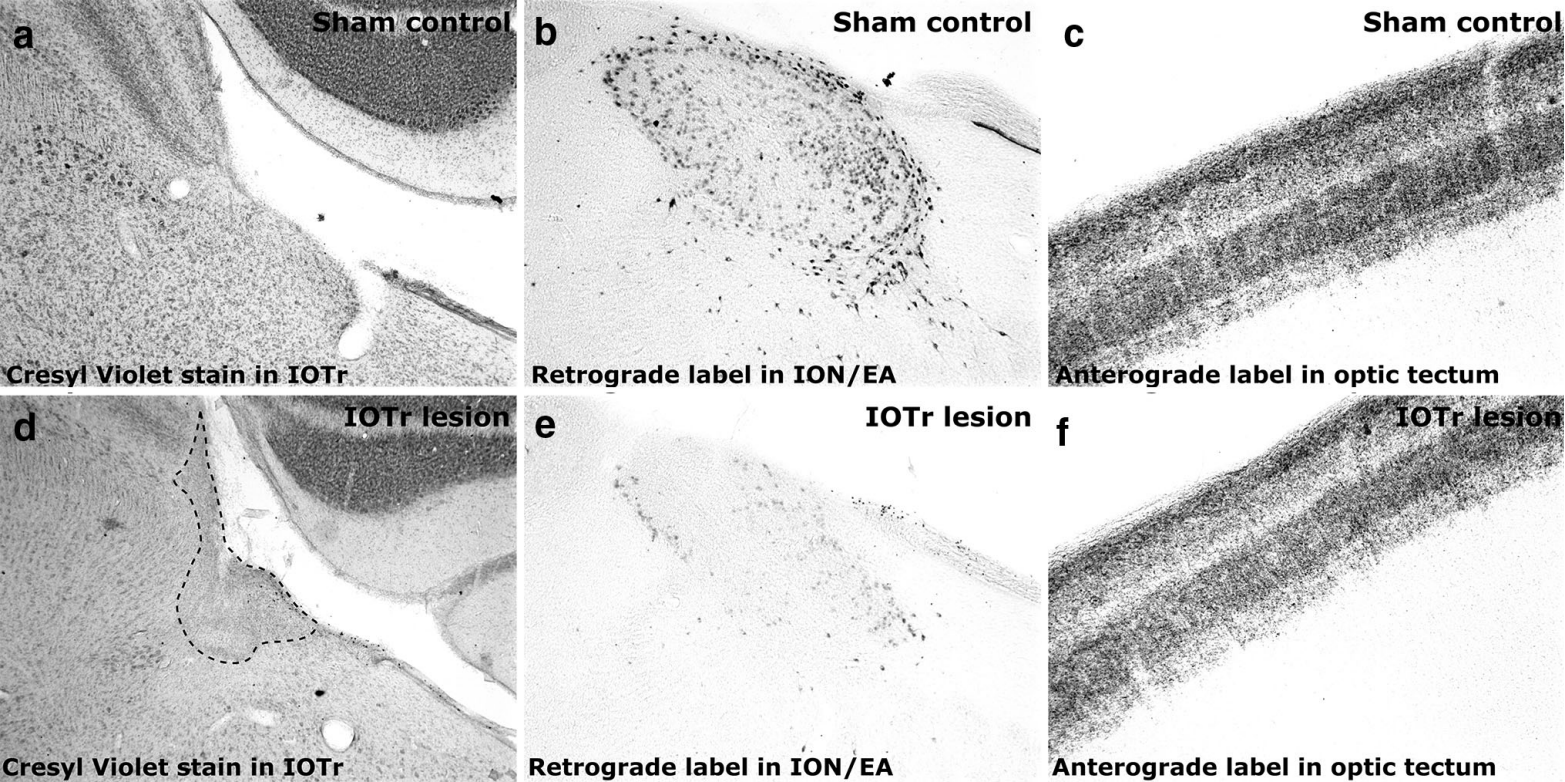
Cresyl Violet stained sections of a sham control case (**a**) and an isthmo-optic tract lesion case (IOTr; **d**). *Dashed lines* in **d** delineate the boundary of the IOTr lesion; **b**, **e** Retrograde wheatgerm agglutinin label in sham and IOTr lesion cases, respectively, at comparable anterior–posterior positions in the hindbrain. Retrograde label in the lesion case (**e**) is substantially reduced relative to the retrograde label in the sham control case (**b**); **c**, **f** Anterograde wheatgerm agglutinin label in the dorsolateral optic tectum (contralateral to the injected eye) of sham (**c**) and lesion cases (**f**), respectively. Comparably dense anterograde label clearly demonstrates tracer efficacy in each case. Abbreviations: EA, ectopic area; ION, isthmo-optic nucleus

One group of chicks (*n* = 8) was used to assess the normal, strain-specific baseline ocular growth pattern under constant light conditions (*n* = 8), i.e., these chicks did not undergo surgery to disrupt centrifugal efferents, for comparison with the ocular growth pattern of chicks of the same strain raised under normal diurnal light conditions (14 h light, 10 h dark cycle; data taken from a control group of 14 chicks in a previous, related study; Dillingham et al. 2013). Subsequently, a second age-matched group (*n* = 17) either underwent stereotaxic surgery to perform a unilateral electrolytic lesion of the IOTr or ION, in order to disrupt centrifugal neurons to the retina (*n* = 12; Fig. 2), or a sham procedure in which the electrode was lowered to the same coordinates but no current was passed (*n* = 5; Fig. 2). Given the uncertainty regarding the pathway by which ipsilateral ectopic centrifugal neurons reach their target, we made the simplifying assumption that the axons of such cells simply remain uncrossed at the optic chiasm. In this scenario, a unilateral lesion of the IOTr would result in the ipsilateral retina receiving intact contralateral ION and EA efferents but not ipsilateral EA efferents, while the contralateral retina would receive only ipsilateral EA efferents (Fig. 1). Details of the surgical approach used have been described previously (Dillingham et al. 2013).

### Measurements

For details of the ocular phenotyping techniques used, please refer to Prashar et al. (2009). Briefly, refractive state (REF) was measured on fully awake chicks using non-cycloplegic streak retinoscopy at 7 and 21 days post-surgery. High-frequency A-scan ultrasonography (20 MHz) was also performed on anaesthetized chicks at 21 days post-surgery to measure ocular component dimensions: ACD, anterior chamber depth; LT, lens thickness; VCD, vitreous chamber depth; and AXL, axial length.

### Determination of lesion success

Damage to the IOTr or ION caused by the lesion was intended to disrupt centrifugal neurons that were innervating the contralateral retina. After phenotyping at 21 days post-surgery, an intravitreal injection of unconjugated wheatgerm agglutinin (WGA; L-1020, Vector Labs, UK) was administered in the contralateral eye 48 h prior to perfusion, to label all residual ION and EA neurons in their entirety. The degree of induced disruption could then be assessed quantitatively through a comparison of the number of labeled CVS neurons in lesioned animals with the mean number of neurons labeled in the intact CVS of sham-operated subjects. Using this method, the degree of success of each lesion was expressed as the percentage of centrifugal neurons that were disconnected/ablated. Details of the immunohistochemical and histological methodology used have been described previously (Dillingham et al. 2013).

A “1-in-3 series” of brain sections from each subject was traced and neuronal counts were made of the absolute number of retrogradely WGA labeled neurons in each section using a Leica microscope (Model DM6000B, Leica Microsystems GmbH, Wetzlar, Germany), in conjunction with Stereo Investigator software (Version 8; MBF Bioscience, Edinburgh, UK). Cell counts for contralaterally projecting ION and EA centrifugal neurons were made separately and then subtracted from the mean cell counts of ION and EA populations, respectively, from sham-operated subjects. The relative loss in ION and EA cell number was expressed as a percentage of the centrifugal neuron population destroyed. As injections of WGA were made only in the contralateral eye, it was not possible to calculate percentage lesion success for the ipsilateral EA projection.

Rather than estimating the actual number of retinopetal neurons that were present following electrolytic lesion of the IOTr, the cell counts were used to calculate the relative proportion of surviving retinopetal cells as compared to the number of retinopetal neurons that are typically present in brains with no lesion. All tissue sections were processed using identical histological and immunohistochemical protocols. Thus, there was no need to use an unbiased stereological approach, nor were any corrections made (e.g., to account for neuron fragmentation), as would be required to determine the “actual” true number of neurons. Rather, the focus was placed on the consistent identification of retrogradely labeled cells after a lesion and comparing the number found in lesioned cases with those in normal brains. This was achieved through counting directly from the stained sections, an approach that eliminates many of the limitations inherent in post hoc analysis of images, e.g., a fixed field of view, a set focal depth and a set resolution. Similarly, no attempt was made to interpolate the counts from sections that were not reacted immunohistochemically, i.e., in the Cresyl Violet and backup series. All proportions were then calculated from counts made from control/sham and experimental/lesion sections of the same thickness, interval and staining.

In analyzing the effects of lesions, the mean within-animal, i.e., inter-ocular difference, for each ocular component was calculated by subtraction of the ipsilateral eye measurements from contralateral eye measurements. In all subjects, the eye contralateral to the lesion of ION/IOTr was the right eye, and the eye ipsilateral to the lesion was the left eye.

### Data analysis

All graphical and statistical analyses were carried out using the statistical software RStudio: Integrated development environment for R (Version 0.98.1091 [Computer software] Boston, MA; freely available from http://www.rstudio.org/; used in conjunction with R, version 3.2.0 ‘Full of Ingredients’; freely available from http://www.r-project.org/). Independent sample *t* tests were used for comparison of the means of the pooled right and left eye refractive states (REF) and the ocular component dimensions of chicks raised under constant light with those raised under normal diurnal light conditions. For statistical modeling, the CRAN package ‘*lme4*’ (Bates et al. 2015; http://cran.r-project.org/web/packages/lme4) was used, while all graphs were generated using the ‘*ggplot2’* CRAN package (Wickham 2009; http://cran.R-project.org/web/packages/ggplot2). A primary analysis of lesion effect on ocular measurements was performed using the Success_ION+EA_ independent variable (i.e., the mean of combined ION and EA percentage lesion success). Linear regression models were generated to identify the degree to which Success_ION+EA_ explained the observed variability in both absolute ocular measurements as well as ocular component and refractive asymmetry between the two eyes, i.e., anisometropia. In the case of REF data, which had been taken at multiple time points, linear mixed effects models (LME) were generated, to account for repeated measures nature of the observations. Stepwise addition of the categorical fixed effects term, Days post-surgery (Days PS), the interaction term Success_ION+EA_ × Days post-surgery, and the random term Subject ID was used to investigate the direction and magnitude of their associations with the dependent variables, anisometropia and the absolute refractive error in the eye contralateral to the lesion REF_contra_ or ipsilateral to the lesion REF_ipsi_. In the absence of repeated measures for ultrasonography data, the contribution of the fixed effect Success_ION+EA_ to ocular component dimension asymmetry (i.e., ΔACD, ΔLT, ΔVCD, ΔAXL) was assessed by generating linear regression models. In both cases, i.e., REF and ocular component dimension analyses, model selection was based on the Akaike information criterion (AIC), a measure describing the explained deviance given the remaining degrees of freedom of a model, and direct comparison of models prior to, and following addition of, a given fixed effect (Akaike 1974). In addition, optimal LME model design was further confirmed by likelihood ratio tests, i.e., the isolation of the significance of a fixed effect based on a comparison of nested models.

The contributions of ION and EA disconnection to post-surgery changes in ocular component dimension measurements were assessed through a comparison of the explained variance, i.e., the adjusted r-squared value of parallel models containing either Success_ION_ or Success_EA_, respectively. Similarly, but taking into account the repeated measures element of REF measurements, a comparison of the regression coefficients (*B*) of parallel LME models containing either Success_ION_ or Success_EA_ as fixed effect variables was performed. In addition, the relative contribution of either Success_ION_ or Success_EA_ to the goodness of fit of the model was assessed through a direct comparison between a model containing either Success_ION_ or Success_EA_ and a baseline model containing only the random effect, Subject ID, and an intercept of 1. Data are presented as mean ± SD unless otherwise stated, while error bars on graphs represent 95 % confidence intervals. A *p* value of <0.05 was considered statistically significant.

All experimental procedures involving animals complied with the U.K. Home Office legislation and the European Communities Council Directive 86/609/EEC (1986).

## Results

### IOTr lesions

**Fig. 3:**
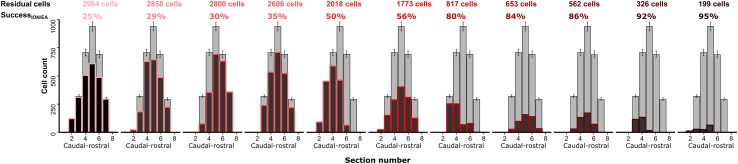
A sequence of histograms showing the distribution, along the caudal–rostral sections of the rostral hindbrain (*1*–*8*, respectively), of remaining retrogradely labeled retinopetal neurons, across a range of lesion success (25–95 %). Superimposed in gray on each plot is the distribution and variance (95 % confidence interval) of retrogradely labeled neurons in sham isthmo-optic tract lesioned cases

A clear reduction in retrogradely labeled cells was evident in the contralateral hemisphere of cases with lesions of the isthmo-optic tract (IOTr) (Fig. 2). Lesions of the IOTr ranged in percentage success from 25 to 95 % (2984–199 remaining cells, respectively; Fig. 3).

### Constant light effect in unoperated chicks

We compared the refractive error (REF) and ocular component dimensions of unoperated chicks raised under either constant light or diurnal light conditions (Table 1). For these comparisons, the average readings of the right and left eye were calculated (after it had been established that there was no significant anisometropia, or fellow-eye ocular component dimension asymmetry, between the two groups; Table 1). No significant difference in REF was observed between the constant light vs. diurnal light groups; however, a highly significant relative ACD shortening (*t*_13.511_ = −17.755, *p* < 0.001) and lens thinning (*t*_19.883_ = −4.332, *p* < 0.001) were evident in the constant light group relative to the diurnal light group. In addition, highly significant VCD elongation was observed in constant light-reared chicks (*t*_16.191_ = −5.302, *p* < 0.001), presumably as a result of visually guided compensation for the constant light-induced ACD and LT changes. The combination of anterior segment shortening and VCD elongation in constant light-reared birds, compared to their diurnal light counterparts, resulted in AXL being similar between the two groups of chicks (*t*_−18.931_ = −1.663, *p* = 0.113).

**Table 1:** Normal light vs. constant light effect on ocular phenotype in unoperated chicks

Absolute phenotyping measurements	Normal light (NL) (±SD) (*n* = 14)	Constant light (CL) (±SD) (*n* = 8)	NL-CL (±SE)
Refractive state (D)	2.27 ± 0.55	2.58 ± 0.98	0.31 ± 0.31
Anterior chamber depth (mm)	1.716 ± 0.060	1.079 ± 0.101	−0.637 ± 0.033***
Lens thickness (mm)	2.475 ± 0.057	2.374 ± 0.056	−0.101 ± 0.023***
Vitreous chamber depth (mm)	5.987 ± 0.209	6.530 ± 0.272	0.544 ± 0.098***
Axial length (mm)	10.178 ± 0.274	9.983 ± 0.288	−0.194 ± 0.116

Mean absolute ocular measurements for sham-operated chicks, comparing those raised under normal diurnal conditions (Q1 chicks from a previous study (Dillingham et al. 2013)) and those raised in constant light

Statistical test results relate to within-group analyses (i.e., is contralateral eye different to ipsilateral eye) * p < 0.05, ** p < 0.01, *** p < 0.005 (independent sample *t* tests)

**Table 2:** The relative contribution of ION or EA lesion success to the effect size observed in Anisometropia, Vitreous Chamber Depth (VCD) Asymmetry (T-C) and the absolute VCD in ipsilateral and contralateral eyes

Dependent variable	Predictors	*B*	*t*	*Λ*
Anisometropia (D)	ION: week	−0.033	−3.091	*χ* ^2^ = 9.400 (*p* = 0.002***)
	EA: week	−0.034	−3.099	*χ* ^2^ = 9.442 (*p* = 0.002***)
ΔVCD (mm)	ION	0.003 ± 0.001	3.474	0.003***
	EA	0.002 ± 0.001	2.422	0.029*
VCD ipsilateral (mm)	ION	−0.005 ± 0.002	−3.086	0.008**
	EA	−0.006 ± 0.001	−4.495	<0.001***
VCD contralateral (mm)	ION	−0.002 ± 0.002	−1.037	0.316
	EA	−0.004 ± 0.002	−2.175	0.046*

The regression coefficient (*B*), *t* statistic, and the degree to which addition of the specified predictors contributed to the goodness of fit when compared with a baseline model containing the random effect, Subject ID, and an intercept of 1 (*Λ*), as defined by the *χ* ^2^ statistic and the *P* value (*p*)

### Anisometropia in constant light chicks with IOTr lesion

**Fig. 4:**
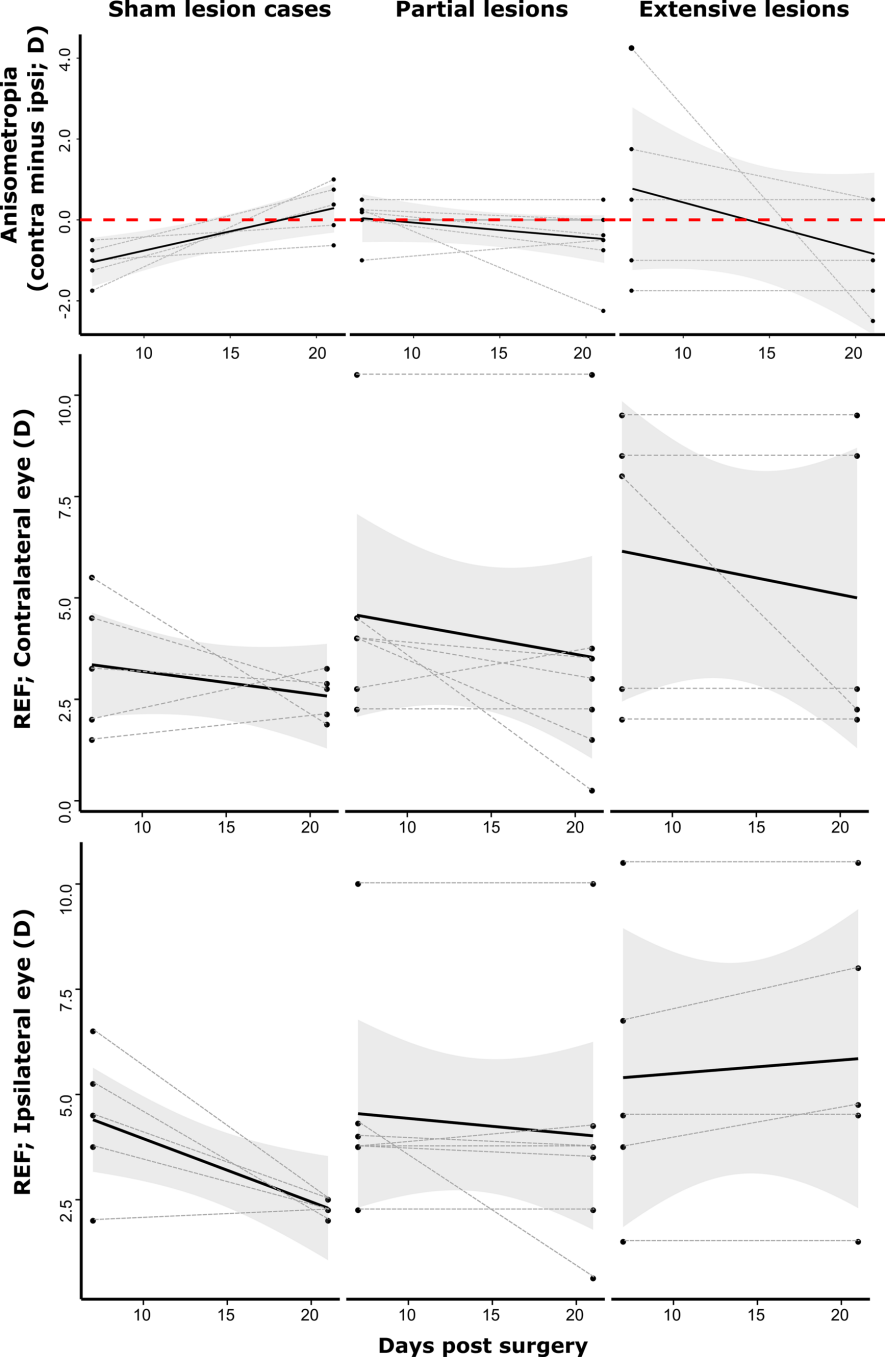
Change in anisometropia (*top panel*), contralateral eye refractive state (*middle panel*) and ipsilateral eye refractive state (*lower panel*) over the course of the experimental time period. Sham-lesioned cases all showed a reduction in anisometropia and refractive state towards isotropia and emmetropia, respectively. Lesion cases have been arbitrarily split into partial (*n* = 7; range 25–56 %) and extensive (*n* = 5; range 80–95 %) lesion cases. The variance in anisometropia and refractive state in both eyes increased considerably in chicks with extensive lesions while high hyperopia was frequently observed in both eyes, often persisting throughout the 21 days post-surgery time period, suggesting a compromised ability to emmetropize. Gray dashed lines denote the progression of individual cases over time while solid black lines and shaded gray regions show linear relationships and 95 % confidence intervals, respectively

Any anisometropia that was present in chicks that had received a sham lesion at 7 days post-surgery had resolved within ±1 D by 21 days post-surgery with all cases showing a change towards a correction of the initial ametropia, a situation mirrored by 6 of the 7 ‘partial’ IOTr-lesioned chicks (*n* = 7; range 25–57 %; Fig. 4). The exception, which had the largest lesion in the partial lesion range (57 %), ended the experimental period with −2.25 D of anisometropia. Of the chicks with ‘extensive’ lesions (*n* = 5, range 80–95 %), anisometropia was only reduced in one chick (92 % lesion success), although in this case the level of anisometropia had been reduced through an increase in ipsilateral eye hyperopia (at 21 days post-surgery, the ipsilateral and contralateral eyes were +8.0 D and +8.5 D, respectively).

**Fig. 5:**
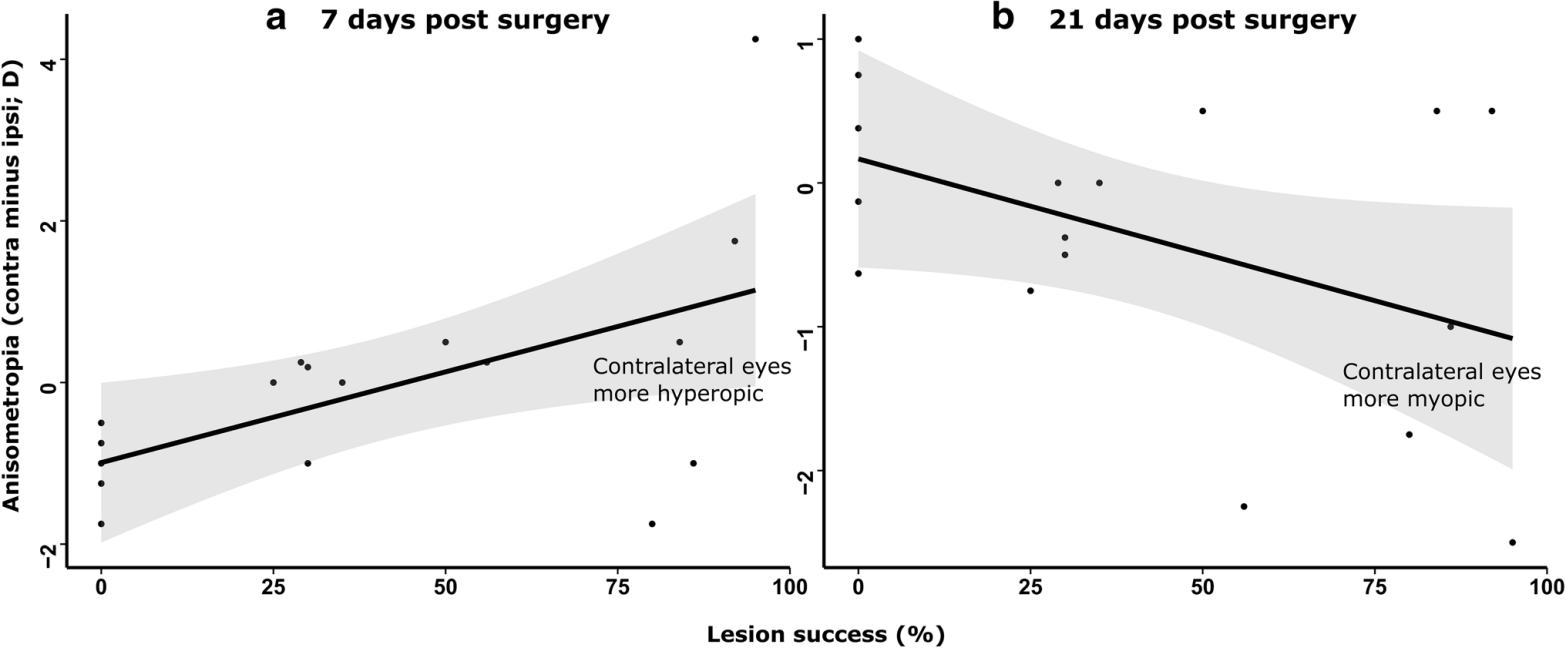
Anisometropia (contralateral minus ipsilateral eye refractive state) at 7 days post-surgery (**a**) and 21 days post-surgery (**b**) time points, plotted against percentage ION and EA lesion success [Success_ION+EA_]. At 7 days post-surgery, a significant positive association between anisometropia and lesion success was evident. By 21 days post-surgery, this association had reversed, exhibiting a negative trend with lesion success. Shaded regions around trend lines represent 95 % confidence intervals

A subsequent linear mixed effects regression analysis revealed a positive association between anisometropia (expressed as the REF in the contralateral eye minus that in the ipsilateral eye) and increasing lesion success, Success_ION+EA_, at 7 days post-surgery (*F*_(1,15)_ = 6.698, *p* = 0.021; Fig. 5a). By 21 days post-surgery, however, this association had reversed, tending instead towards a negative association (*F*_(1,15)_ = 3.929, *p* = 0.066; Fig. 5b). Reflecting this temporal reversal of this relationship, inclusion of the interaction term Success_ION+EA_ × Days PS (in addition to Success_ION+EA_, Days PS and Subject ID) significantly improved the fit of a linear model in which anisometropia was the dependent variable (interaction term *B* = −0.036 ± 0.011, *t* = −3.260, *p* = 0.001). This interaction effect manifested the transition from relative hyperopia in the contralateral eye at 7 days post-surgery to relative myopia in the contralateral eye at 21 days post-surgery in chicks with highly successful CVS lesions.

### Absolute refractive state in constant light chicks with IOTr lesion

By 21 days post-surgery, as expected, the refractive states of the contralateral and ipsilateral eyes of all sham lesioned cases had reduced from 3.9 ± 1.7 D at 7 days post-surgery to be tightly clustered around 2.5 D (2.4 ± 0.4 D) by day 21 (Fig. 4). While the majority of the contralateral eyes of the partial IOTr lesion cases showed a similar trend towards emmetropia over the experimental time frame (6 of 7 chicks), there was one exception (with a 50 % lesion success) that exhibited high hyperopia (which was mirrored in the ipsilateral eye; 10.0 D and 10.5 D, respectively), which persisted to day 21. Of the extensive lesion cases, both the contralateral and ipsilateral eyes of 2 of the 5 chicks (86 and 92 % lesion success) showed similarly high hyperopia (8.8 ± 1.6 D) at 7 days post-surgery, which remained stable, or had increased by day 21 (9.1 ± 1.1 D; Fig. 4). The chick with the highest lesion success (95 %) showed high hyperopia in the contralateral eye (8.0 D) combined with a nearly emmetropic ipsilateral eye (3.75 D) at 7 days post-surgery, i.e., considerable anisometropia (+4.25 D). By day 21, the previously hyperopic contralateral eye had emmetropized, while the ipsilateral eye had become more hyperopic (4.75 D), again resulting in considerable anisometropia (−2.5 D).

**Fig. 6:**
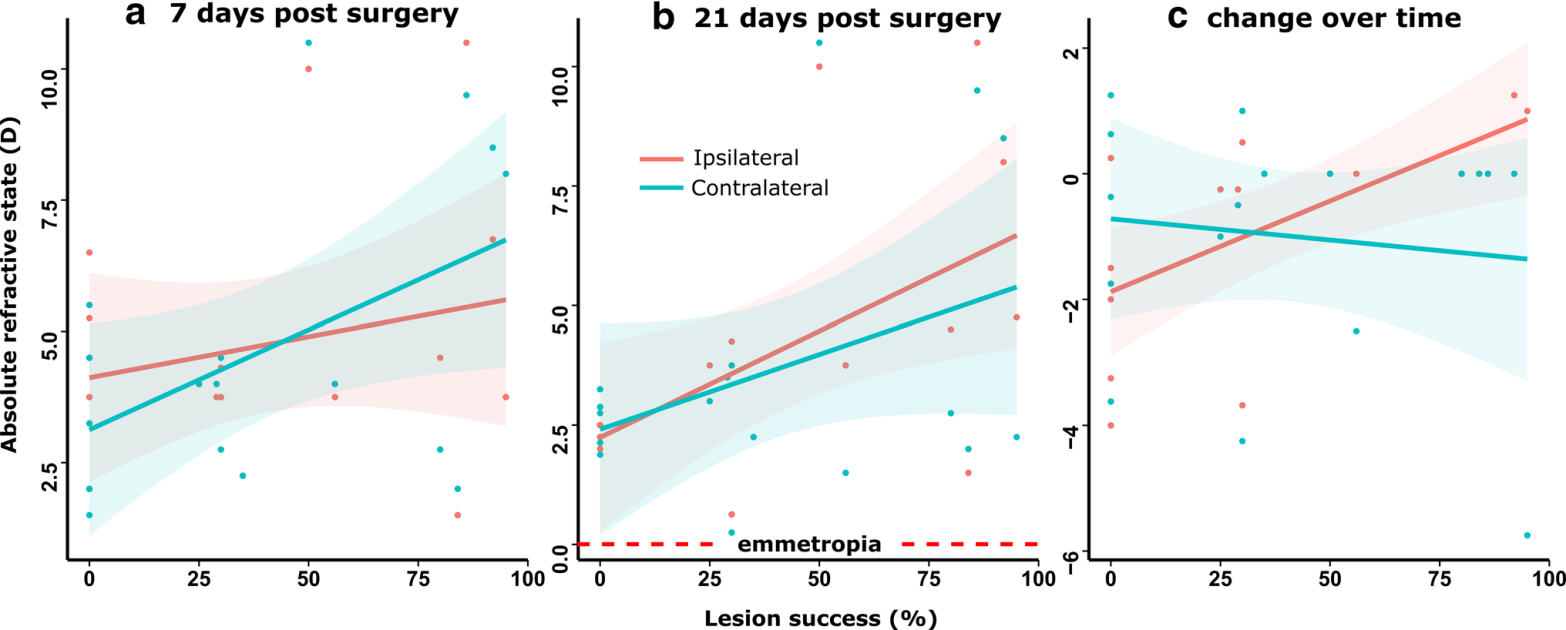
Absolute refractive errors of eyes ipsilateral (*blue*) and contralateral (*red*) to the isthmo-optic nucleus/tract lesion at 7 (**a**) and 21 (**b**) days post-surgery and the change in refractive state in ipsilateral and contralateral eyes associated with lesion success (**c**). At 7 days post-surgery, the refractive state of eyes contralateral to the lesion showed a significant positive correlation with lesion success while the eye ipsilateral to the lesion did not. Conversely, 21 days post-surgery, the refractive states of the eyes contralateral to the lesion did not exhibit a significant trend with lesion success whereas the refractive states of the ipsilateral eyes did, with the degree of hyperopia increasing significantly with lesion success. During the course of the experimental time period, ipsilateral eyes showed a significant positive association with lesion success, whereas contralateral eyes did not, suggesting that lesion-associated changes during this time were predominantly the result of a hyperopic shift in the ipsilateral eyes

Analyses analogous to that described above for anisometropia were performed to gauge the effects of IOTr lesions on the absolute refractive error of each eye: REF_contra_, the eye contralateral to the lesion, and REF_ipsi_, the eye ipsilateral to the lesion. These revealed a significant positive association between Success_ION+EA_ and REF_contra_ at 7 days post-surgery (*F*_1,15_ = 4.62, *R* ^2^ = 0.185, *p* < 0.05), while no association was evident in a parallel analysis of REF_ipsi_ (Fig. 6a). Conversely, by 21 days post-surgery, REF_ipsi_ exhibited a significant positive relationship with Success_ION+EA_ (*F*_1,15_ = 6.648, *R* ^2^ = 0.261, *p* < 0.05), while contralateral eyes did not (*R* ^2^ = 0.090; Fig. 6b).

Including the refractive state variance of individual chicks during the observation period revealed a positive association between REF_contra_ and Success_ION+EA_ (*B* = + 0.035 ± 0.017, *t* = 2.010, *p* = 0.045), combined with a negative association between REF_contra_ and days post-surgery (*B* = −0.992 ± 0.474, *t* = −2.091, *p* = 0.038). The latter finding reflects a shift from a transient lesion-associated relative hyperopia at 7 days post-surgery towards emmetropia in contralateral eyes by 21 days post-surgery. Crucially, it was notable that, while the fit of a mixed linear model for REF_ipsi_ was significantly improved by the addition of a Success_ION+EA_ × days PS interaction term (*B* = 0.029 ± 0.009, *t* = 3.254, *p* = 0.001), this was not the case for the best-fit model for REF_contra_ (*B* = −0.007 ± 0.014, *t* = 0.480, *p* = 0.599), suggesting that lesion-associated changes in refractive state between 7 and 21 days post-surgery were predominantly the result of a hyperopic shift in the ipsilateral eyes (Fig. 6c). Finally, increased lesion success was significantly correlated with the variability in measurements observed in refractive state at 7 and 21 days post-surgery, in both contralateral (*t*_4_ = 3.653, *r* = 0.837, *p* = 0.022) and ipsilateral eyes (*t*_4_ = 3.868, *r* = 0.888, *p* = 0.018).

### Ocular component dimensions changes in constant light chicks with IOTr lesion

**Fig. 7:**
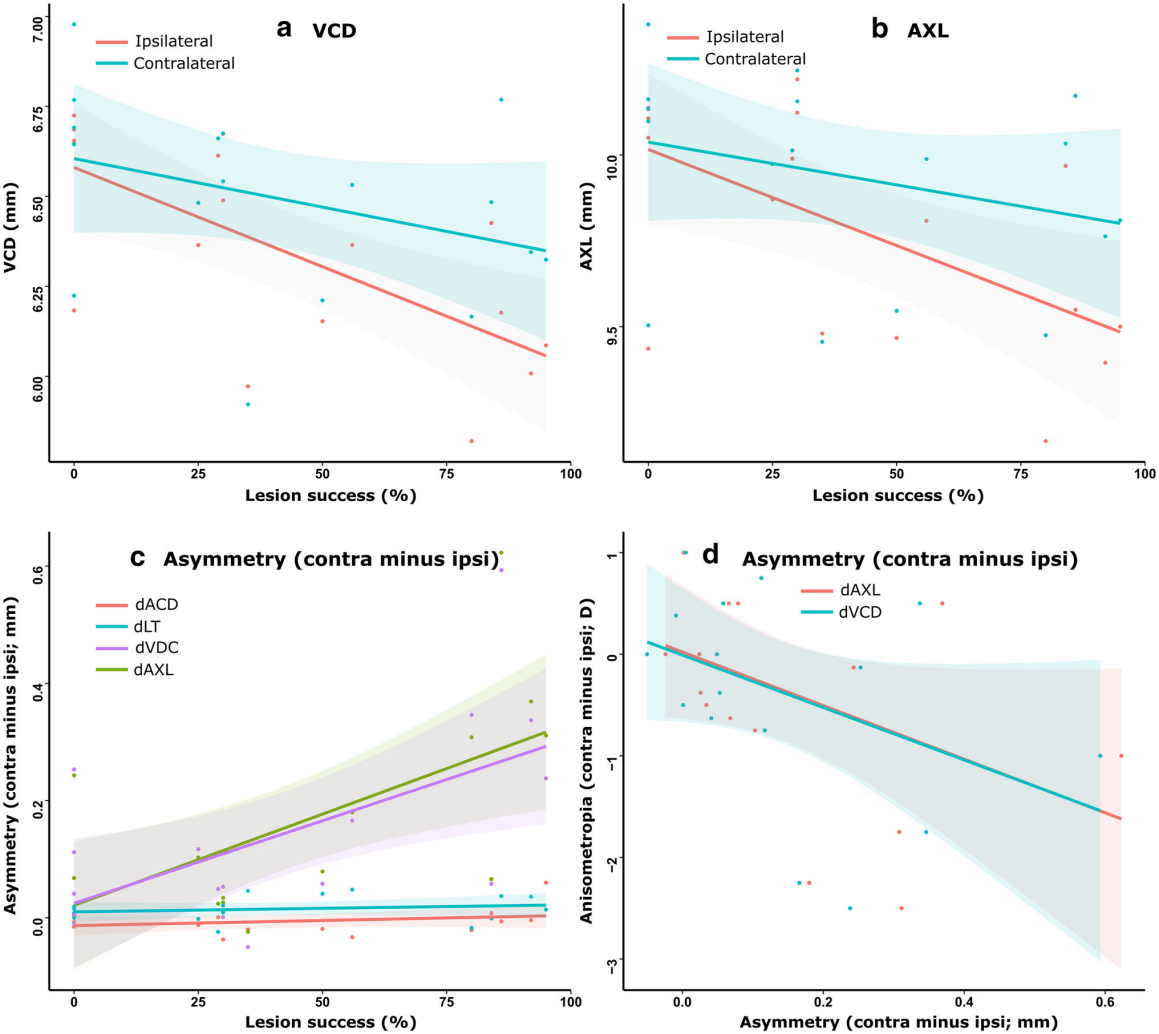
Vitreous chamber (**a**) and axial length (**b**) dimensions of contralateral (*blue*) and ipsilateral (*red*) eyes at 21 days post-surgery, plotted against percentage lesion success (Success_ION+EA_). No significant association was observed between the VCD or AXL of contralateral eyes, while ipsilateral eye VCD and AXL both exhibited significant associations with lesion success. Significant positive associations between lesion success and both ΔVCD and ΔAXL were observed (**c**), corresponding to a reduction in VCD and AXL in ipsilateral eyes relative to contralateral eyes, with increasing lesion success, while anterior chamber depth (*red*) and lens thickness asymmetry (*blue*) showed no association with increasing lesion success. Both VCD and AXL asymmetry (**d**) were significantly negatively correlated with anisometropia, suggesting that the observed anisometropia was vitreous chamber dependent. Shaded regions around trend lines represent 95 % confidence intervals of variance

No lesion-associated trends in anterior chamber depth or lens thickness asymmetry were evident at 21 days post-surgery. Similarly, there was no significant association between the absolute dimensions of any of these components with Success_ION+EA_ in either contralateral or ipsilateral eyes (Fig. 7c). However, there was a significant positive association between ΔVCD and Success_ION+EA_ (*F*_1,15_ = 8.488, *R* ^2^ = 0.319, *p* = 0.011) at 21 days post-surgery (Fig. 7c), and this was mirrored by a similar relationship between ΔAXL and Success_ION+EA_ (*F*_1,15_ = 10.51, *R* ^2^ = 0.373, *p* = 0.005), reflecting a trend towards relative axial elongation of contralateral eyes with increasing percentage lesion success (Fig. 7c). Both ΔVCD and ΔAXL showed strong negative associations with anisometropia (*F*_1,32_ = 7.300, *R* ^2^ = 0.16 *p* = 0.011), suggesting that the observed lesion-associated changes were vitreous chamber dependent (Fig. 7d). Linear models in which the absolute VCD of contralateral (VCD_contra_) or ipsilateral (VCD_ipsi_) eyes was fitted as the dependent variable revealed a significant negative association between VCD_ipsi_ and Success_ION+EA_ (*F*_1,15_ = 12.700, *R* ^2^ = 0.423, *p* < 0.005) but no relationship between VCD_contra_ and Success_ION+EA_ (Fig. 7a). Analogous results were seen for axial length (AXL_ipsi_: *F*_1,15_ = 8.237, *R* ^2^ = 0.311, *p* < 0.05; AXL_contra_: *p* > 0.05; Fig. 7b). Thus, when compared with the vitreous chamber elongation characteristic of normal constant light rearing (i.e., in unoperated eyes), disruption of centrifugal neurons was correlated with a reduced relative axial elongation in ipsilateral eyes.

### Isthmo-optic nucleus (ION)/ectopic area (EA)

The complexity of the centrifugal projection is such that each eye receives 3 projections: contralateral ION efferents, contralateral EA efferents and ipsilateral EA efferents. Although lesion success, as defined by the proportion of ION neurons destroyed, was strongly correlated with EA lesion success (*ρ* = 0.918, *df* = 15, *t* = 8.946, *p* < 0.001), the level of accuracy with which lesion success was quantified in this study, i.e., using neuronal tracers, enabled the treatment effects to be analyzed as a function of the degree to which each population of contralateral ION or contralateral EA neurons was disrupted by the lesion. As only unilateral intravitreal injections of WGA were made, it was not possible to quantify the lesion success in the ipsilateral EA projection. Such an approach provides some insight into whether disconnection of one population of centrifugal neurons contributed to a greater or lesser extent than the other to the observed ocular lesion effects. The results suggest that ION and EA lesion success contributed more or less equally to lesion associated anisometropia (Table 2); however, loss of ION, rather than contralateral EA neurons, was more strongly correlated with ΔVCD. A subsequent linear analysis of ION and EA lesion success with the absolute measurements for both contralateral and ipsilateral eye VCD revealed that EA lesion success exhibited a stronger negative association than ION lesion success. Both ION and EA lesion success were significantly correlated with the observed variance in VCD_ipsi_, EA lesion success showing a stronger relationship. In addition, only EA lesion success was significantly correlated with the variance observed in VCD_contra_.

**Table 3:** Comparison of the effects of surgical, e.g., optic nerve section or ciliary ganglion section, and optical manipulations upon emmetropization, in chicks raised under constant light and normal diurnal conditions

Manipulation	Changes compared to normal phenotype under		References
	Normal light	Constant light	
Optic nerve section	Hyperopia, reduced VCD	No effect	Troilo et al. (1987); Troilo and Wallman (1991); Li and Howland (2000); Wildsoet (2001)
Ciliary ganglion section	Hyperopia, corneal flattening, reduced VCD	Hyperopia, reduced ACD, corneal flattening, reduced VCD	Li and Howland (2000); Wildsoet (2003)
Hyperopic defocus	Normal compensation	Exaggerated hyperopia	Bartmann et al. (1994); Wildsoet (2003)
Myopic defocus	Normal compensation	Hyperopic shift	Troilo and Wallman (1991); Bartmann et al. (1994)

## Discussion

In contrast to findings in White Leghorn chicks (Li et al. 1995), high hyperopia was not a feature of constant light rearing in unoperated birds of the dark-feathered Shaver Black strain used here (Table 3). Thus, although some unoperated chicks did develop a degree of hyperopia commensurate with that seen in previous work (i.e., +8.00 to +10.00 D), the remaining chicks were either isometropic with, or more myopic than, age-matched chicks raised under diurnal lighting. Unlike unoperated chicks, birds with unilateral disruption of centrifugal neurons raised under constant light developed a transient hyperopic shift in the eye contralateral to the lesion. Thus, contralateral eyes were more hyperopic than ipsilateral eyes 7 days post-surgery, but not 21 days post-surgery. In contrast, ipsilateral eyes showed a lesion-induced hyperopia at 21 days post-surgery that was not evident at the earlier 7 days post-surgery time point (Fig. 6).

The finding of a transient, initial hyperopia in the contralateral eyes of chicks with a highly successful ION/IOTr lesion concurs with our findings in ION/IOTr-lesioned chicks raised under normal diurnal lighting (Dillingham et al. 2013). In the present constant light study, by the end of the 21-day observation period, contralateral eyes had developed the same ocular phenotype as found in unoperated chicks, i.e., anterior chamber shortening and vitreous chamber elongation. More surprisingly, the degree of centrifugal disruption was significantly correlated with relative axial shortening in ipsilateral eyes by the end of the 21-day follow-up period.

From prior work, the ocular phenotype resulting from constant light is known to be characterized by hyperopia, corneal flattening, increased intra-ocular pressure (IOP), reduced anterior chamber depth, lens thinning and vitreous chamber elongation (Bartmann et al. 1994; Li et al. 1995; Wahl et al. 2015). These effects on the eyes are principally due to the disruption of circadian rhythms (Weiss and Schaeffel 1993) and the concomitant absence of the normal diurnal fluctuations in dopamine and melatonin levels (Parkinson and Rando 1983). In the present study, chicks raised in constant light conditions developed some but not all of these features: reduced anterior chamber depth, lens thinning and vitreous chamber elongation were present, while hyperopia was not. This lack of hyperopia appeared to be because the constant light-induced anterior chamber shortening was matched by commensurate VCD elongation, suggesting an emmetropization response to the reduced power of a flatter cornea. Presumably, our results for constant light rearing may differ from those of other investigators either because of inter-strain variability (e.g., heavy versus light pigmentation) and/or differences in experimental conditions, e.g., light intensity.

Interestingly, primates raised under constant light do not exhibit an ocular phenotype comparable to that in chicks (Smith et al. 2001). Instead, ocular development is normal albeit with the suggestion of increased incidence of refractive anomalies, including myopia and slight axial anisometropias, implying that the emmetropization process may be affected, but to a far lesser degree than observed in the chicken. In other ways, ocular development in both chick and primate models has shown a great degree of commonality under a number of other experimental conditions (Yinon 1984; Smith et al. 1999, 2009). Nevertheless, in the case of constant light rearing, it does not appear that findings in the chick can be extrapolated to the epidemiology of human ametropias (Smith et al. 2001). Thus, while our findings are, perhaps, only relevant to mechanisms of emmetropization in the chick model, the significance of understanding the limitations of the relevance of results obtained using lower vertebrate animal models to higher vertebrates cannot be overstated. Primates have a far less well-established centrifugal projection (Gastinger et al. 2006) than the chick, with distinct anatomical and neurochemical and characteristics that are, if at all, more comparable to the ectopic centrifugal projection in birds. One possibility is that the functionality achieved through tectal feedback to the retina in birds is achieved, in primates, through cortical feedback to visual thalamic nuclei. Indeed, it is interesting to note that those bird species with a more diffuse CVS organization, i.e., without an isthmo-optic nucleus, tend to have a residual population of ectopic neurons (Gutierrez-Ibanez et al. 2012). Such variations in the size and/or degree of organization in the CVS, both within and between orders, are likely to be critical to establishing its function.

The refractive state of contralateral eyes (REF_contra_ *)* of IOTr lesioned chicks, measured at 7 days post-surgery, showed a significant positive relationship with Success_ION+EA_, a feature analogous to that observed in lesioned chicks raised under diurnal light conditions, in which disruption of centrifugal neurons caused an initial transient hyperopic shift in contralateral eyes at 7 days post-surgery that had resolved by 21 days post-surgery (Dillingham et al. 2013). In chicks raised under constant light conditions, the VCD of ipsilateral eyes, i.e., ipsilateral to the IOTr lesion, exhibited a significant negative relationship with Success_ION+EA_, i.e., high lesion success was correlated with reduced VCD. In a previous study (Dillingham et al. 2013), we have discussed possible explanations for this effect. Briefly, we proposed that the initial intra-retinal effect of the lesion was one of transient hyperactivity of the nitric oxide producing target cells of ION neuron projections (IOTCs), resulting from their partial deafferentation (i.e., diaschisis; Stavraky 1961; Sharpless 1964; von Monakow 1969). We proposed that this may have caused a short-term increase in the rate of nitric oxide release. Such an effect would result in the inhibition of normal vitreous chamber elongation and, thus, explain the observed vitreous chamber-dependent hyperopia. Whatever the mechanism for this transient hyperopia, the focus of this discussion will be on the unexpected finding that, by 3 weeks post-surgery, the ipsilateral eyes of chicks showed a significant trend towards becoming shorter with increased IOTr lesion success Success_ION+EA_ (Fig. 7).

Our findings are best considered in the context of optic nerve section (ONS) emmetropization studies (Table 3), as severance of the optic nerve not only transects retinal efferent axons, but also centrifugal neurons to the retina. Under diurnal light conditions, chicks with ONS develop moderate hyperopia in the affected eye (Troilo et al. 1987). To our knowledge, only one study has investigated the combined effects of constant light treatment and ONS surgery on emmetropization (Li and Howland 2000). In that study, chicks were raised under constant light for 7 days before undergoing unilateral ONS surgery. Four weeks following surgery, both contralateral and ipsilateral eyes showed no differences from previously reported constant light effects in chicks with intact optic nerves (Li et al. 1995). Specifically, no significant refractive or ocular component dimension asymmetry was observed. In addition to the differences in experimental time points, the constant light + ONS study of Li and Howland and the present study differ in two important ways. Firstly, our experimental paradigm did not disrupt retinal efferent axons in the optic nerve. By lesioning the IOTr, only ~10,000 centrifugal efferent axons (~0.5 % of the total number of optic nerve fibers) were targeted, deafferenting only the small proportion of retinal neurons that receive projections from/are innervated by the CVS. Secondly, while ONS surgery effects are confined to the contralateral eye, IOTr or ION lesions are likely to disrupt pathways to both eyes as a result of the ipsilateral EA pathway. Thus, the complex pattern of lesion-associated changes reported here might be a consequence of both of these pathways being disconnected, raising the possibility that the bilateral centrifugal projections are a requirement for consensual, i.e., symmetric, refractive development. It is worthy of note that direct retino-retinal connections have been shown to exist, e.g., Avellaneda-Chevrier et al. (2015) and Nadal-Nicolás et al. (2015). While such connections could, in theory, underlie the effects described here, their significance is unclear and they are, in all likelihood, too few in number to sustain any physiological influence.

An ipsilateral eye effect was evident from the significant negative association that was observed between VCD_ipsi_ and Success_ION+EA_ for individual subjects (*F*_1,15_ = 12.720, *p* = 0.003), while only a much weaker, non-significant negative correlation existed between VCD_contra_ and percentage lesion success (*F*_1,15_ = 2.203, *p* = 0.158), again consistent with an ipsilateral eye effect. These data raise the possibility that both contralateral and ipsilateral centrifugal pathways may act to regulate emmetropization at some level, perhaps via independent pathways. It is, therefore, clear that, with hindsight, a limitation of this study was the inability to quantify the degree to which the ipsilateral EA projection was disrupted by the lesion of the IOTr. A number of emmetropization studies have reported fellow untreated eye effects following unilateral experimental paradigms. For example, changes in ZENK expression were found predominantly in ipsilateral eyes following unilateral plus or minus lens wear (Bitzer and Schaeffel 2002). Furthermore, as mentioned above, when chicks were form deprived unilaterally, the normal oscillatory diurnal growth (i.e., growth during the day and slight shrinkage at night) of the ipsilateral eye was also disrupted, and although the eye remained emmetropic, growth was consistently slow and did not exhibit a diurnal pattern (Weiss and Schaeffel 1993). Such experiments, like the present study, provide evidence that a reciprocal connection between the eye and the brain is necessary for normal ocular growth patterns associated with circadian rhythms. Moreover, our results offer additional support for the hypothesis that symmetrical growth of fellow eyes is dependent on inter-ocular communication, presumably either through systemic, i.e., endocrine, responses to visual stimuli, or through cross-talk between higher visual centers, such as the CVS, given its bilateral projection to the two eyes.

The neurochemical properties of IOTCs have been well established as exhibiting nitric oxide synthase and NADPH-diaphorase immunoreactivity (Morgan et al. 1994; Fischer and Stell 1999; Wilson and Lindstrom 2011). Indeed, these findings, in part, formed the basis for our proposed interpretation of previous findings in CVS-lesioned chicks raised under diurnal light (Dillingham et al. 2013), given that nitric oxide has been implicated in ocular growth mechanisms (Fujikado et al. 1997; Nickla et al. 2009). On the other hand, equivalent neurochemical data are not available for the target cells of ectopic centrifugal neurons, including the subpopulation that projects to the ipsilateral retina. Under normal diurnal light conditions, disruption of centrifugal neurons in the same strain of chicks implicated isthmo-optic, rather than ectopic, centrifugal neurons in the observed anisometropia (characterized by transient, contralateral eye hyperopia; Dillingham et al. 2013). In the present study, although no difference in the strength of association between ION or EA lesion success and anisometropia was evident, ION lesion success explained a greater amount of the variance in ΔVCD than EA lesion success. In contrast, and of particular interest, while both were significant, the absolute measurements for VCD in ipsilateral eyes (at 21 days post-surgery) showed a stronger, negative association with EA, rather than ION, lesion success (Table 2). The general consensus that ipsilaterally projecting centrifugal neurons to the retina are derived from the ectopic neuronal population, combined with their implication in the ipsilateral eye effect described here, raises the possibility that ectopic centrifugal neurons play a role in circadian rhythm-driven modulation of emmetropization mechanisms.

As the electrophysiological and neurochemical mechanisms of the ectopic centrifugal projection have not been elucidated, any mechanistic explanation for the observed ipsilateral eye hyperopia in the context of the ipsilateral centrifugal pathway would only be speculative. Instead, we will consider two biogenic amines, melatonin and dopamine, in the broader context of our results. First, on the one hand, melatonin is likely to be a critical factor in the development of the ocular phenotype associated with constant light rearing. Endocrine melatonin synthesis by the pineal gland is light dependent. Chicks reared under constant light conditions, but wearing hoods that shield the pineal gland from the surrounding illumination, do not develop the same anterior chamber characteristics (Li and Howland 2006). Similarly, chemical destruction of the retina does not disrupt corneal growth rates (Oishi et al. 1996). Thus, it is likely that systemic, rather than local, melatonin mechanisms are responsible for the observed anterior chamber effects.

In the present study, disruption of centrifugal neurons did not result in an altered anterior chamber phenotype, suggesting that the observed CVS lesion-associated posterior chamber effects were presumably the consequence of disrupted local retinal mechanisms (i.e., the absence of CVS input), rather than a compensation for corneal or lenticular changes. On the other hand, dopamine has similarly been implicated in the mechanisms responsible for the axial elongation associated with form deprivation (Bartmann et al. 1994). Unilateral form deprivation results in the bilateral disruption of diurnal patterns of ocular growth (Weiss and Schaeffel 1993), i.e., in both contralateral and ipsilateral eyes. In addition, dopamine and DOPAC (a metabolite of dopamine) levels are reduced following both form deprivation (Bartmann et al. 1994) and constant light rearing. In both cases, the reduction was between 30 and 40 % and peaked following 8–14 days of either treatment. In the context of our findings, the delay between the onset of a constant light treatment and the point at which dopamine levels are significantly reduced is suggestive, given that the major changes in eye growth rates reported here took place during this time. In addition, dopamine may be an important explanatory factor as a result of its reported interactions with retinal nitric oxide in the bovine retina. Nitric oxide antagonists were shown to increase endogenous dopamine levels, while nitric oxide generators reduced retinal dopamine levels (Bugnon et al. 1994).

A recent study suggests dopamine and nitric oxide mechanisms are interdependent in the chick retina, with dopamine seemingly acting upstream of nitric oxide (Nickla et al. 2013). In that study, inhibition of axial elongation in response to hyperopic defocus or form deprivation, through treatment with dopamine agonists, was prevented by intravitreal injection of nitric oxide antagonists, i.e., inhibition of nitric oxide led to disinhibition of vitreous chamber elongation. Thus, in the present study, a potential mechanistic explanation for the additional influence of constant light on emmetropization following disruption of the CVS is that constant light-induced increased dopamine levels occur simultaneously with lesion-induced reductions in nitric oxide release, resulting in the observed inhibition of vitreous chamber elongation. However, this does not provide an explanation for the temporal pattern of changes observed first in the contralateral eye and then in the ipsilateral eye.

In summary, under constant light conditions, unilateral disruption of centrifugal ION and EA neurons (and/or their axons) projecting to the retina of the contralateral eye induced an initial, transient hyperopia in the contralateral eye, which had resolved 2 weeks later, in a manner similar to the lesion-induced changes that have previously been reported under normal diurnal conditions. However, under constant light conditions, centrifugal disruption induced relative axial hyperopia in the eye ipsilateral to the lesion, which persisted for at least the 21-day duration of the experiment. This finding implicates ipsilaterally projecting centrifugal EA neurons in the regulation of emmetropization mechanisms, perhaps via nitric oxide/dopaminergic pathways. The complexity of ametropias associated with CVS lesions in chicks under different lighting conditions suggests that the CVS may play an important role in regulating a light-dependent lateralization of development. Indeed, the rudimentary nature of the CVS in mammals might help explain the fact that constant light rearing in primate models only produces mild ametropias, if any.
